# Crystal structure of 4-eth­oxy-*N*-(4-eth­oxy­phen­yl)-*N*-phenyl­aniline

**DOI:** 10.1107/S160053681401900X

**Published:** 2014-08-30

**Authors:** Liang-Tao Wu, Ming Kong, Jie-Ying Wu

**Affiliations:** a Deparment of Chemistry, Anhui University, Hefei 230601, People’s Republic of China; b Key Laboratory of Functional Inorganic Materials Chemistry, Hefei 230601, People’s Republic of China

**Keywords:** crystal structure, tri­phenyl­amine derivatives, supra­molecular chains, C—H⋯π inter­actions

## Abstract

In the title compound, C_22_H_23_NO_2_, the planes of the eth­oxy­benzene rings are oriented with respect to that of the phenyl ring at dihedral angles of 61.77 (8) and 84.77 (8)°, and they are twisted with respect to one another, with a dihedral angle of 80.37 (7)°. In the crystal, weak C—H⋯π inter­actions link the mol­ecules into supra­molecular chains propagating along [101].

## Related literature   

For applications of tri­phenyl­amine derivatives, see: Liu *et al.* (2012 ▶); Pina *et al.* (2013 ▶). For related compounds, see: Wang *et al.* (2011 ▶); Gudeika *et al.*(2012 ▶). For properties of triphenyl derivatives, see: Costa & Santos (2013 ▶); Metri *et al.* (2012 ▶).
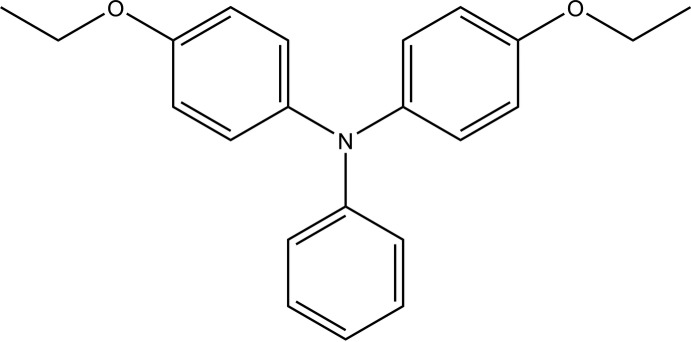


## Experimental   

### Crystal data  

C_22_H_23_NO_2_
*M* *_r_* = 333.41
Monoclinic, 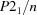

*a* = 7.3634 (7) Å
*b* = 31.908 (3) Å
*c* = 8.1372 (8) Å
β = 107.598 (1)°
*V* = 1822.4 (3) Å^3^
*Z* = 4
Mo *K*α radiation
μ = 0.08 mm^−1^
*T* = 298 K
0.30 × 0.20 × 0.20 mm

### Data collection  

Bruker APEXII CCD diffractometer
13155 measured reflections
3274 independent reflections
2288 reflections with *I* > 2σ(*I*)
*R*_int_ = 0.035

### Refinement  

*R*[*F* ^2^ > 2σ(*F* ^2^)] = 0.042
*wR*(*F* ^2^) = 0.106
*S* = 1.03
3274 reflections
228 parameters
H-atom parameters constrained
Δρ_max_ = 0.13 e Å^−3^
Δρ_min_ = −0.16 e Å^−3^

### 

Data collection: *APEX2* (Bruker, 2007 ▶); cell refinement: *SAINT* (Bruker, 2007 ▶); data reduction: *SAINT*; program(s) used to solve structure: *SHELXTL* (Sheldrick, 2008 ▶); program(s) used to refine structure: *SHELXTL*; molecular graphics: *SHELXTL*; software used to prepare material for publication: *SHELXTL*.

## Supplementary Material

Crystal structure: contains datablock(s) I, Global. DOI: 10.1107/S160053681401900X/xu5812sup1.cif

Structure factors: contains datablock(s) I. DOI: 10.1107/S160053681401900X/xu5812Isup2.hkl

Supporting information file. DOI: 10.1107/S160053681401900X/xu5812Isup3.cml

**Fig. 1.**
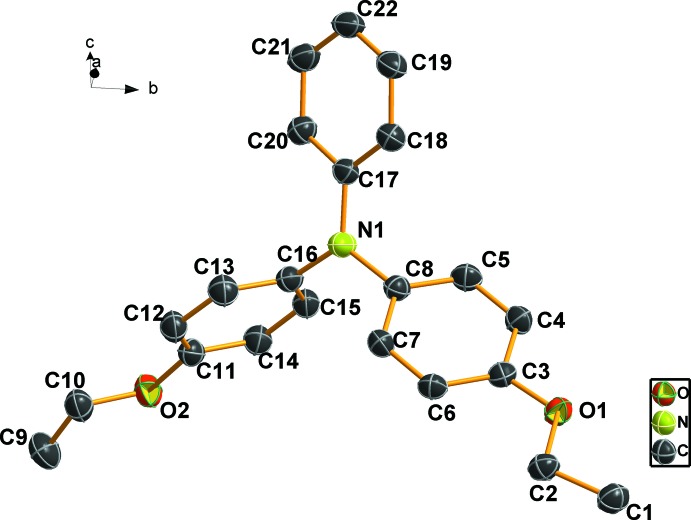
The molecular structure of the title compound showing 30% probability displacement ellipsoids.

. DOI: 10.1107/S160053681401900X/xu5812fig1.tif
The mol­ecular structure of the title compound showing 30% probability displacement ellipsoids.

**Fig. 2.**
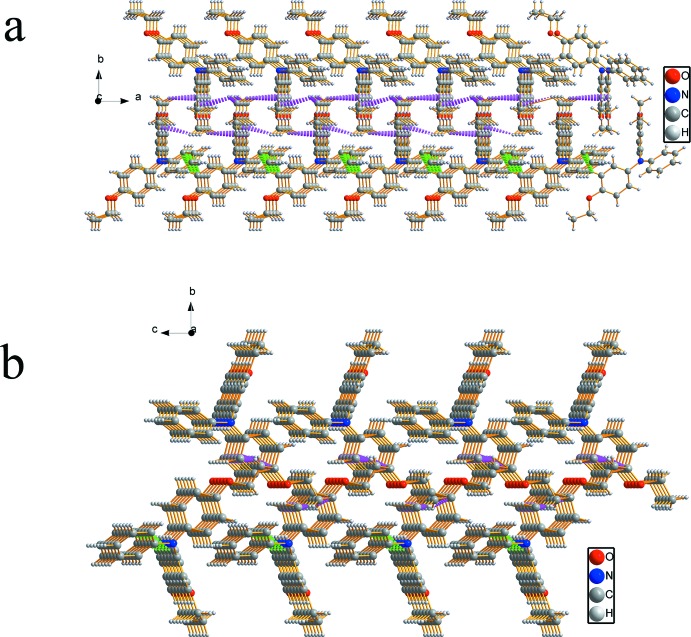
The weak interactions among molecules.

. DOI: 10.1107/S160053681401900X/xu5812fig2.tif
The weak interactions among molecules.

CCDC reference: 1016997

Additional supporting information:  crystallographic information; 3D view; checkCIF report

## Figures and Tables

**Table 1:** Hydrogen-bond geometry (Å, °) *Cg*1 is the centroid of the C3–C8 ring.

*D*—H⋯*A*	*D*—H	H⋯*A*	*D*⋯*A*	*D*—H⋯*A*
C1—H1*A*⋯*Cg*1^i^	0.96	2.83	3.6763 (17)	148

Symmetry code: (i) 
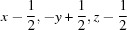
.

## supplementary crystallographic information

### S1. Comment

Tripheylamine derivatives catch considerable interest and attention in application of OLEDs and efficient optical chemosensors due to useful properties in electrical conductivity and electroluminescence (Pina *et al.*, 2013; Liu *et al.*, 2012). Much effort has been made to explore the relationship between their structures and properties. The ethoxyl groups as donors in the title compound have enhanced the properties in several optical applications, its special structure also contributes to its transport properties when used to those areas (Costa & Santos, 2013 and Metri *et al.*, 2012). In the molecule, the two ethoxybenzene rings are oriented with respect to the phenyl ring at 61.77 (8) and 84.77 (8)°, and they are twisted to each other with a dihedral angle of 80.37 (7)°. In the crystal, weak C—H···π interaction links the molecules into the supramolecular chains propagated along the [101] direction.

### S2. Experimental

A mixture of 4-iodophenol and sodium hydroxide was grinded for 0.5 h and added to 1000 ml flask, following the addition of bromoethane (750 ml) as solvent, Cs_2_CO_3_ (4 g) and 18-crown-6 (1 g, 3.78 mmol) as catalysts. The mixture was refluxed for 72 h, and obtained yellow oil was washed with NaOH solution (500 ml, 5%) until neutral. After extraction with dichloromethane (50 ml) for three times, the organic solution was evaporated, which yielded the intermediate as a white product (101 g, 90.5%). A 1,2-dichlorobenzene (purified) solution containing synthesized 4-ethoxy-iodobenzene (20.86 g, 75 mmol), aniline (2.38 g, 22 mmol), K_2_CO_3_ (17.94 g, 130 mmol), Cu powder (8.34 g, 130 mmol), 18-crown-6 (100 mg, 0.38 mmol) was stirred under N_2_ for 0.5 h at room temperature, refluxed for 2 h, and continuous reaction at air. After cooling, copper was filtered out, 1,2-dichlorobenzene was evaporated, then white solid was obtained through column chromatography purification. 1H NMR: (400 MHz, (C_1_D_3)_2_C_1_O_1), d(p.p.m.): d(p.p.m.): 7.18–7.14 (t, 2H), 7.02–7.00 (d, 4H), 6.86–6.71 (m, 7H), 4.04–3.99 (m, 4H), 1.37–1.34 (t, 6H).

### S3. Refinement

All hydrogen atoms were placed in geometrically idealized positions and constrained to ride on their parent atoms, with C—H = 0.93–0.97 Å, *U*_iso_(H) = 1.2*U*_eq_(C) or 1.5*U*_eq_(C).

### Crystal data

**Table:**

C_22_H_23_NO_2_	*F*(000) = 712
*M**_r_* = 333.41	*D*_x_ = 1.215 Mg m^−^^3^
Monoclinic, *P*2_1_/*n*	Mo *K*α radiation, λ = 0.71073 Å
Hall symbol: -P 2yn	Cell parameters from 2593 reflections
*a* = 7.3634 (7) Å	θ = 2.5–21.4°
*b* = 31.908 (3) Å	µ = 0.08 mm^−^^1^
*c* = 8.1372 (8) Å	*T* = 298 K
β = 107.598 (1)°	Block, colorless
*V* = 1822.4 (3) Å^3^	0.30 × 0.20 × 0.20 mm
*Z* = 4	

### Data collection

**Table:**

Bruker APEXII CCD diffractometer	2288 reflections with *I* > 2σ(*I*)
Radiation source: fine-focus sealed tube	*R*_int_ = 0.035
Graphite monochromator	θ_max_ = 25.2°, θ_min_ = 2.6°
phi and ω scans	*h* = −8→8
13155 measured reflections	*k* = −38→36
3274 independent reflections	*l* = −9→9

### Refinement

**Table:**

Refinement on *F*^2^	Primary atom site location: structure-invariant direct methods
Least-squares matrix: full	Secondary atom site location: difference Fourier map
*R*[*F*^2^ > 2σ(*F*^2^)] = 0.042	Hydrogen site location: inferred from neighbouring sites
*wR*(*F*^2^) = 0.106	H-atom parameters constrained
*S* = 1.03	*w* = 1/[σ^2^(*F*_o_^2^) + (0.0539*P*)^2^ + 0.0295*P*] where *P* = (*F*_o_^2^ + 2*F*_c_^2^)/3
3274 reflections	(Δ/σ)_max_ = 0.001
228 parameters	Δρ_max_ = 0.13 e Å^−^^3^
0 restraints	Δρ_min_ = −0.16 e Å^−^^3^

### Special details

**Table:**

Geometry. All esds (except the esd in the dihedral angle between two l.s. planes) are estimated using the full covariance matrix. The cell esds are taken into account individually in the estimation of esds in distances, angles and torsion angles; correlations between esds in cell parameters are only used when they are defined by crystal symmetry. An approximate (isotropic) treatment of cell esds is used for estimating esds involving l.s. planes.
Refinement. Refinement of F^2^ against ALL reflections. The weighted R-factor wR and goodness of fit S are based on F^2^, conventional R-factors R are based on F, with F set to zero for negative F^2^. The threshold expression of F^2^ > 2sigma(F^2^) is used only for calculating R-factors(gt) etc. and is not relevant to the choice of reflections for refinement. R-factors based on F^2^ are statistically about twice as large as those based on F, and R- factors based on ALL data will be even larger.

### Fractional atomic coordinates and isotropic or equivalent isotropic displacement parameters (Å^2^)

**Table:**

	*x*	*y*	*z*	*U*_iso_*/*U*_eq_	
N1	0.56747 (19)	0.12535 (4)	0.45337 (16)	0.0565 (4)	
O1	0.53889 (15)	0.25318 (3)	−0.02262 (14)	0.0552 (3)	
O2	−0.07739 (15)	0.02297 (3)	0.28521 (16)	0.0634 (3)	
C1	0.5355 (2)	0.28483 (5)	−0.2863 (2)	0.0621 (5)	
H1A	0.4353	0.3026	−0.2738	0.093*	
H1B	0.5177	0.2798	−0.4064	0.093*	
H1C	0.6563	0.2982	−0.2358	0.093*	
C2	0.5312 (2)	0.24385 (5)	−0.1966 (2)	0.0543 (4)	
H2A	0.4153	0.2287	−0.2540	0.065*	
H2B	0.6393	0.2267	−0.1985	0.065*	
C3	0.5385 (2)	0.22017 (5)	0.08506 (19)	0.0441 (4)	
C4	0.5504 (2)	0.23063 (5)	0.2528 (2)	0.0510 (4)	
H4	0.5517	0.2587	0.2843	0.061*	
C5	0.5603 (2)	0.19973 (5)	0.3734 (2)	0.0530 (4)	
H5	0.5684	0.2072	0.4859	0.064*	
C6	0.5319 (2)	0.17841 (5)	0.0390 (2)	0.0513 (4)	
H6	0.5204	0.1709	−0.0741	0.062*	
C7	0.5424 (2)	0.14783 (5)	0.1615 (2)	0.0522 (4)	
H7	0.5385	0.1198	0.1295	0.063*	
C8	0.5585 (2)	0.15785 (5)	0.33024 (19)	0.0458 (4)	
C9	−0.2755 (3)	−0.03507 (6)	0.1792 (3)	0.0809 (6)	
H9A	−0.3214	−0.0332	0.2775	0.121*	
H9B	−0.2783	−0.0638	0.1429	0.121*	
H9C	−0.3551	−0.0185	0.0869	0.121*	
C10	−0.0747 (2)	−0.01898 (5)	0.2265 (2)	0.0628 (5)	
H10A	0.0083	−0.0362	0.3167	0.075*	
H10B	−0.0282	−0.0197	0.1270	0.075*	
C11	0.0897 (2)	0.04537 (5)	0.32911 (19)	0.0494 (4)	
C12	0.2629 (2)	0.03055 (5)	0.3206 (2)	0.0575 (4)	
H12	0.2738	0.0031	0.2863	0.069*	
C13	0.4208 (2)	0.05683 (5)	0.3634 (2)	0.0591 (5)	
H13	0.5368	0.0470	0.3559	0.071*	
C14	0.0770 (2)	0.08574 (5)	0.3849 (2)	0.0542 (4)	
H14	−0.0388	0.0957	0.3926	0.065*	
C15	0.2339 (2)	0.11136 (5)	0.4291 (2)	0.0541 (4)	
H15	0.2238	0.1384	0.4678	0.065*	
C16	0.4072 (2)	0.09730 (5)	0.41669 (19)	0.0494 (4)	
C17	0.7052 (2)	0.12510 (5)	0.61526 (19)	0.0462 (4)	
C18	0.8721 (2)	0.14857 (5)	0.6470 (2)	0.0536 (4)	
H18	0.8907	0.1656	0.5608	0.064*	
C19	1.0098 (2)	0.14661 (5)	0.8058 (2)	0.0628 (5)	
H19	1.1204	0.1624	0.8248	0.075*	
C20	0.6829 (2)	0.10018 (5)	0.7488 (2)	0.0549 (4)	
H20	0.5727	0.0843	0.7314	0.066*	
C21	0.8226 (3)	0.09876 (6)	0.9064 (2)	0.0637 (5)	
H21	0.8052	0.0819	0.9937	0.076*	
C22	0.9866 (3)	0.12182 (6)	0.9362 (2)	0.0674 (5)	
H22	1.0802	0.1207	1.0426	0.081*	

### Atomic displacement parameters (Å^2^)

**Table:**

	*U*^11^	*U*^22^	*U*^33^	*U*^12^	*U*^13^	*U*^23^
N1	0.0563 (8)	0.0573 (9)	0.0469 (8)	−0.0146 (7)	0.0020 (6)	0.0095 (7)
O1	0.0699 (7)	0.0504 (7)	0.0452 (7)	0.0056 (5)	0.0173 (5)	0.0047 (5)
O2	0.0567 (7)	0.0525 (7)	0.0794 (8)	−0.0090 (6)	0.0184 (6)	−0.0062 (6)
C1	0.0653 (11)	0.0667 (12)	0.0537 (11)	−0.0014 (9)	0.0170 (9)	0.0094 (9)
C2	0.0592 (10)	0.0608 (11)	0.0427 (9)	0.0029 (8)	0.0152 (8)	0.0027 (8)
C3	0.0410 (8)	0.0477 (9)	0.0424 (9)	0.0029 (7)	0.0108 (7)	0.0031 (8)
C4	0.0587 (10)	0.0473 (9)	0.0475 (10)	0.0038 (7)	0.0170 (8)	−0.0043 (8)
C5	0.0613 (10)	0.0586 (11)	0.0406 (9)	−0.0003 (8)	0.0177 (8)	−0.0034 (8)
C6	0.0579 (10)	0.0536 (10)	0.0392 (9)	−0.0026 (8)	0.0100 (7)	−0.0023 (8)
C7	0.0600 (10)	0.0445 (9)	0.0479 (10)	−0.0066 (7)	0.0101 (8)	−0.0050 (8)
C8	0.0428 (9)	0.0493 (10)	0.0418 (9)	−0.0060 (7)	0.0073 (7)	0.0021 (7)
C9	0.0746 (13)	0.0733 (13)	0.0890 (15)	−0.0234 (10)	0.0162 (11)	−0.0096 (11)
C10	0.0723 (12)	0.0518 (11)	0.0646 (12)	−0.0113 (8)	0.0214 (9)	−0.0050 (9)
C11	0.0503 (10)	0.0492 (10)	0.0461 (9)	−0.0065 (7)	0.0108 (7)	0.0033 (7)
C12	0.0615 (11)	0.0440 (9)	0.0662 (12)	−0.0021 (8)	0.0179 (9)	−0.0032 (8)
C13	0.0532 (10)	0.0563 (11)	0.0672 (12)	−0.0002 (8)	0.0175 (9)	−0.0009 (9)
C14	0.0544 (10)	0.0496 (10)	0.0591 (11)	0.0011 (8)	0.0180 (8)	0.0012 (8)
C15	0.0636 (11)	0.0439 (9)	0.0531 (10)	−0.0035 (8)	0.0152 (8)	−0.0006 (7)
C16	0.0510 (9)	0.0508 (10)	0.0414 (9)	−0.0072 (8)	0.0062 (7)	0.0045 (7)
C17	0.0504 (9)	0.0446 (9)	0.0411 (9)	0.0015 (7)	0.0100 (7)	−0.0019 (7)
C18	0.0553 (10)	0.0524 (10)	0.0498 (10)	−0.0034 (8)	0.0107 (8)	−0.0025 (8)
C19	0.0552 (10)	0.0608 (11)	0.0617 (12)	−0.0038 (8)	0.0018 (9)	−0.0080 (9)
C20	0.0575 (10)	0.0569 (10)	0.0497 (10)	−0.0005 (8)	0.0153 (8)	0.0023 (8)
C21	0.0772 (13)	0.0652 (12)	0.0457 (10)	0.0101 (10)	0.0139 (9)	0.0043 (9)
C22	0.0718 (12)	0.0681 (12)	0.0485 (11)	0.0082 (10)	−0.0027 (9)	−0.0077 (9)

### Geometric parameters (Å, º)

**Table:**

N1—C17	1.3985 (19)	C9—H9B	0.9600
N1—C8	1.4296 (18)	C9—H9C	0.9600
N1—C16	1.4384 (18)	C10—H10A	0.9700
O1—C3	1.3705 (17)	C10—H10B	0.9700
O1—C2	1.4312 (18)	C11—C14	1.378 (2)
O2—C11	1.3736 (17)	C11—C12	1.382 (2)
O2—C10	1.4237 (19)	C12—C13	1.389 (2)
C1—C2	1.502 (2)	C12—H12	0.9300
C1—H1A	0.9600	C13—C16	1.376 (2)
C1—H1B	0.9600	C13—H13	0.9300
C1—H1C	0.9600	C14—C15	1.372 (2)
C2—H2A	0.9700	C14—H14	0.9300
C2—H2B	0.9700	C15—C16	1.385 (2)
C3—C6	1.381 (2)	C15—H15	0.9300
C3—C4	1.382 (2)	C17—C18	1.395 (2)
C4—C5	1.378 (2)	C17—C20	1.395 (2)
C4—H4	0.9300	C18—C19	1.382 (2)
C5—C8	1.381 (2)	C18—H18	0.9300
C5—H5	0.9300	C19—C22	1.375 (2)
C6—C7	1.380 (2)	C19—H19	0.9300
C6—H6	0.9300	C20—C21	1.381 (2)
C7—C8	1.379 (2)	C20—H20	0.9300
C7—H7	0.9300	C21—C22	1.372 (2)
C9—C10	1.501 (2)	C21—H21	0.9300
C9—H9A	0.9600	C22—H22	0.9300
			
C17—N1—C8	122.07 (12)	O2—C10—H10A	110.3
C17—N1—C16	120.50 (12)	C9—C10—H10A	110.3
C8—N1—C16	116.40 (12)	O2—C10—H10B	110.3
C3—O1—C2	117.76 (12)	C9—C10—H10B	110.3
C11—O2—C10	118.31 (12)	H10A—C10—H10B	108.5
C2—C1—H1A	109.5	O2—C11—C14	115.30 (14)
C2—C1—H1B	109.5	O2—C11—C12	125.23 (14)
H1A—C1—H1B	109.5	C14—C11—C12	119.48 (14)
C2—C1—H1C	109.5	C11—C12—C13	119.72 (15)
H1A—C1—H1C	109.5	C11—C12—H12	120.1
H1B—C1—H1C	109.5	C13—C12—H12	120.1
O1—C2—C1	107.40 (13)	C16—C13—C12	120.71 (15)
O1—C2—H2A	110.2	C16—C13—H13	119.6
C1—C2—H2A	110.2	C12—C13—H13	119.6
O1—C2—H2B	110.2	C15—C14—C11	120.51 (15)
C1—C2—H2B	110.2	C15—C14—H14	119.7
H2A—C2—H2B	108.5	C11—C14—H14	119.7
O1—C3—C6	125.08 (13)	C14—C15—C16	120.63 (15)
O1—C3—C4	115.74 (13)	C14—C15—H15	119.7
C6—C3—C4	119.16 (14)	C16—C15—H15	119.7
C5—C4—C3	120.33 (15)	C13—C16—C15	118.91 (14)
C5—C4—H4	119.8	C13—C16—N1	121.09 (15)
C3—C4—H4	119.8	C15—C16—N1	119.96 (14)
C4—C5—C8	121.12 (14)	C18—C17—C20	117.78 (14)
C4—C5—H5	119.4	C18—C17—N1	121.23 (14)
C8—C5—H5	119.4	C20—C17—N1	120.95 (14)
C7—C6—C3	119.78 (14)	C19—C18—C17	120.33 (16)
C7—C6—H6	120.1	C19—C18—H18	119.8
C3—C6—H6	120.1	C17—C18—H18	119.8
C8—C7—C6	121.61 (14)	C22—C19—C18	121.37 (17)
C8—C7—H7	119.2	C22—C19—H19	119.3
C6—C7—H7	119.2	C18—C19—H19	119.3
C7—C8—C5	117.96 (14)	C21—C20—C17	120.81 (16)
C7—C8—N1	120.09 (14)	C21—C20—H20	119.6
C5—C8—N1	121.92 (14)	C17—C20—H20	119.6
C10—C9—H9A	109.5	C22—C21—C20	121.00 (17)
C10—C9—H9B	109.5	C22—C21—H21	119.5
H9A—C9—H9B	109.5	C20—C21—H21	119.5
C10—C9—H9C	109.5	C21—C22—C19	118.70 (16)
H9A—C9—H9C	109.5	C21—C22—H22	120.6
H9B—C9—H9C	109.5	C19—C22—H22	120.6
O2—C10—C9	107.15 (14)		

### Hydrogen-bond geometry (Å, º)

Cg1 is the centroid of the C3–C8 ring.

**Table:**

*D*—H···*A*	*D*—H	H···*A*	*D*···*A*	*D*—H···*A*
C1—H1*A*···*Cg*1^i^	0.96	2.83	3.6763 (17)	148

Symmetry code: (i) *x*−1/2, −*y*+1/2, *z*−1/2.
