# Supplementary figures and images for: Crystal structure of 4-eth­oxy-N-(4-eth­oxy­phen­yl)-N-phenyl­aniline
Source: Acta Crystallogr Sect E Struct Rep Online. 2014 Aug 30;70(Pt 9):o1077. doi: 10.1107/S160053681401900X (PMC4186099; doi:10.1107/S160053681401900X)

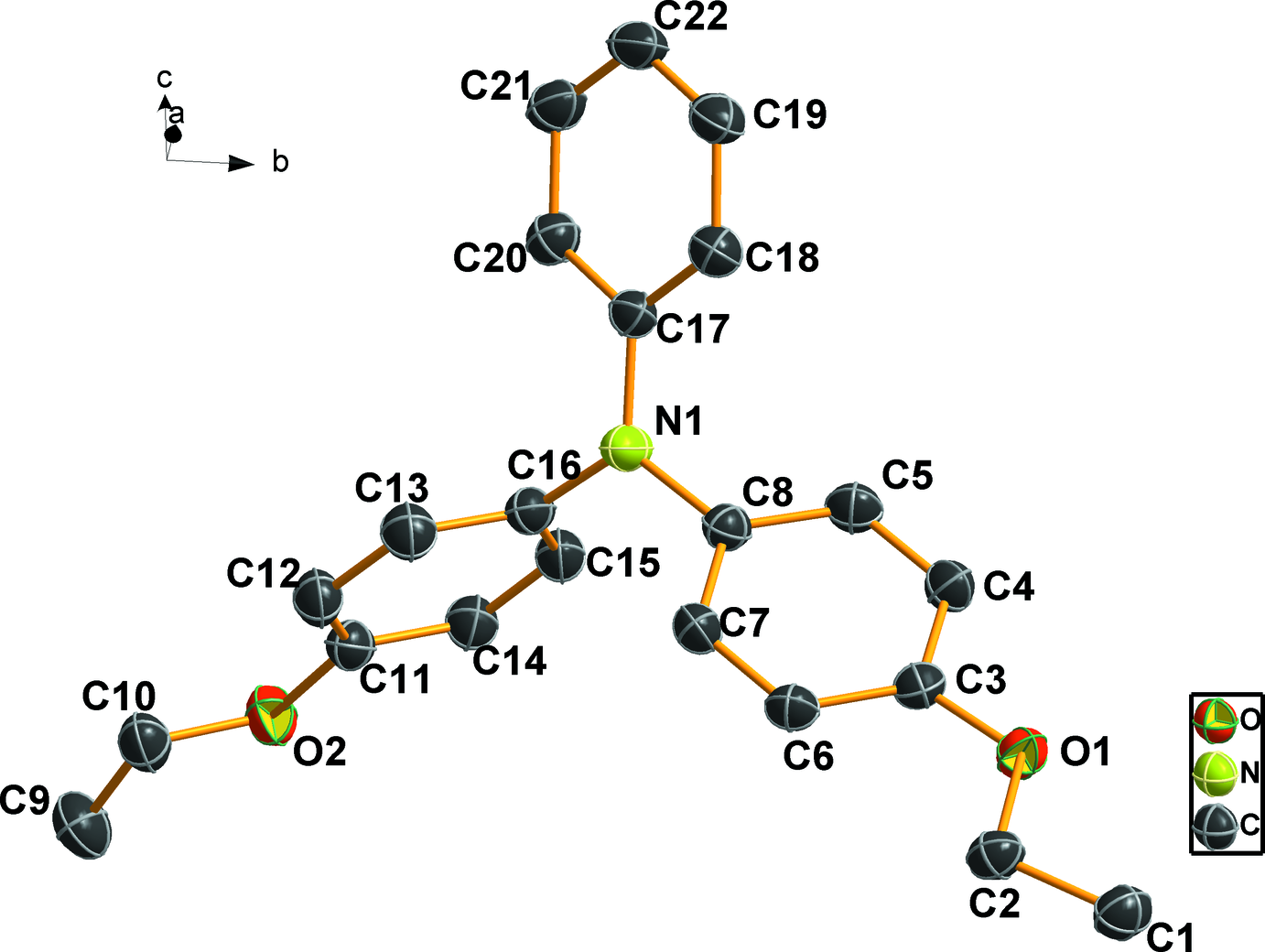

Supplement: Supplementary file 4 [file e-70-o1077-fig1.tif]

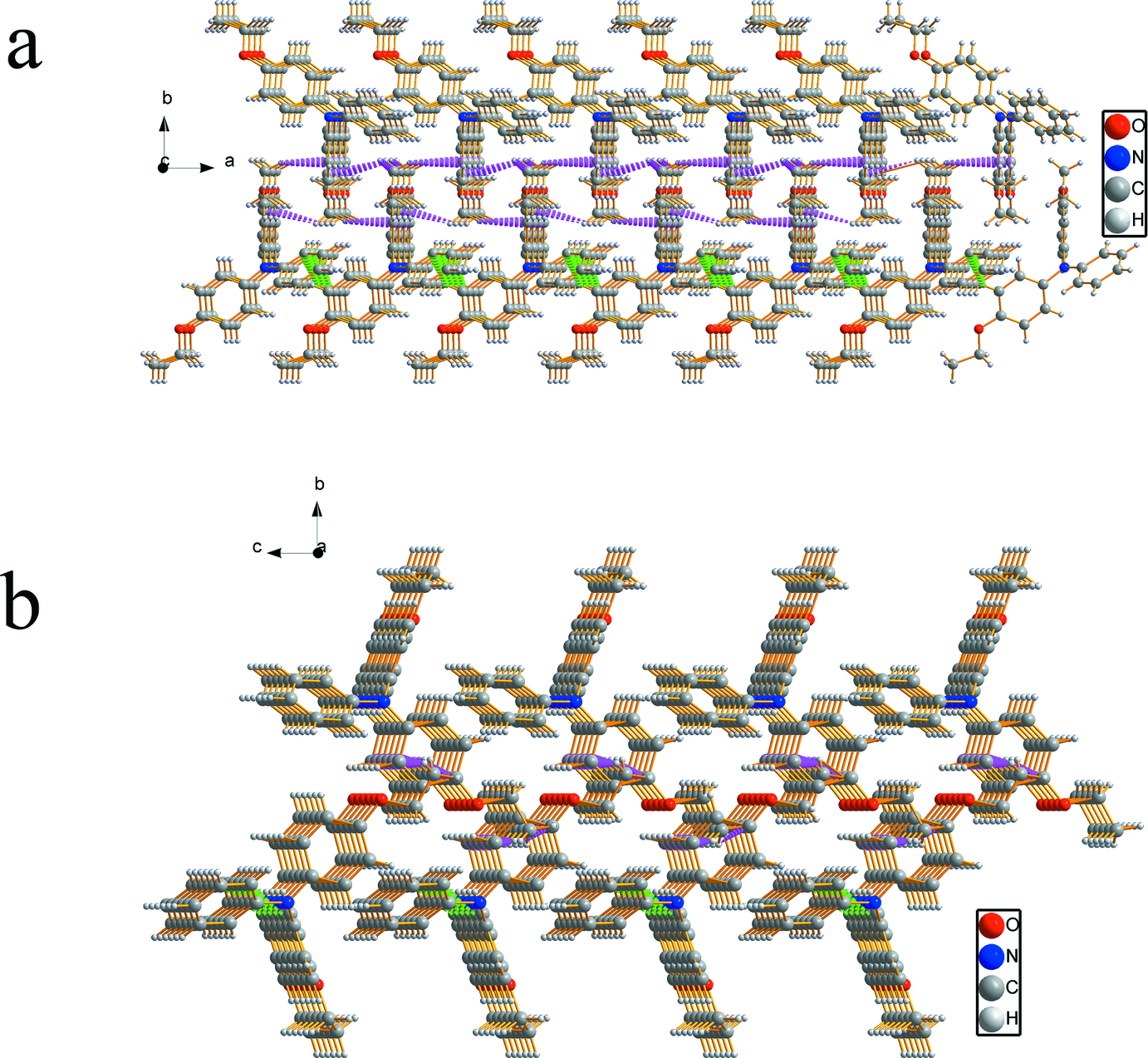

Supplement: Supplementary file 5 [file e-70-o1077-fig2.tif]
